# Understanding the Effects of Emergency Experience on Online First-Aid Learning Intention: The Mediating Role of Psychological Distances and Prosociality

**DOI:** 10.3389/fpsyg.2021.829804

**Published:** 2022-01-20

**Authors:** Shuo Zhang, Huijing Guo, Xiaofeng Ju, Jiantao Ma

**Affiliations:** ^1^Harbin Institute of Technology, Harbin, China; ^2^China National Knowledge Infrastructure (CNKI), Beijing, China

**Keywords:** learning intention, emergency experience, psychological distances, prosociality, first-aid

## Abstract

The fast-paced lifestyle resulting from China's rapid economic development has caused more than 500 million sudden cardiac deaths in 2020. Online first-aid education for the public is considered a key potential solution to avoid such incidents and would have great practical value. This study focuses on understanding the impact of past first-aid experience on the intention for online learning of first-aid knowledge and skills from the perspective of individual psychological factors based on the construction level and prosociality theories. More specifically, it tests the mediating roles of psychological distances and prosociality, which reflect psychological status, in the relationship between experience and learning intention along with a series of associated demographic variables. Primary data collected *via* a survey are analyzed through regression analysis. The results show that first-aid experience has a significant positive impact on online first-aid learning intention. In addition, psychological distances from first-aid events, and prosociality, play mediating roles in the relationship between first-aid experience and learning intention. Thus, this study contributes to understanding first-aid learning intention by revealing the impact of the individual factors of psychological distances and prosociality.

## Introduction

With the rapid development of the social economy, the incidence of sudden diseases is relatively high owing to the fast pace of life and unhealthy lifestyles. Implementing correct and simple emergency rescue immediately after an emergency incidence can effectively reduce the mortality and disability rates caused by sudden diseases (Kanstad et al., 2011). Therefore, it is of great practical significance to popularize first-aid knowledge and skills and promote the population's related knowledge and skills. However, the public in China lacks opportunities to be trained formally in a standardized and systematic manner. In this regard, the online learning of first-aid knowledge and skills based on the development of online learning technology breaks through the limitations of time and space, and thus facilitates training that is more flexible, extensive, and convenient.

Past emergency experience provides an immersive experience for individuals to understand the severe consequence of emergency events. Existing conclusions deducted mixed results to understand the relationship between emergency experience and subsequent behavior. On the one hand, most people with emergency experience generally believe that such an event is unlikely to happen and pay little attention to it (Roppolo et al., 2009), leading to low intention to acquire first-aid knowledge and skills. However, other scholars believed that past emergency experience had a powerful impact on shaping the recognition and judgments about a future emergency (Weinstein, 1989; Martin et al., 2009; Demuth et al., 2016). Results of past studies suggest that past emergency experience is an essential factor to future behavior, such as people's response to the threat (Siegrist and Gutscher, 2008). Learning first-aid knowledge and skills online could help individuals better respond to emergency events in the future. This article aims to understand the relationship between emergency experience and first-aid online learning intention.

It is also important to understand how past experience has an effect on learning the knowledge and skills to deal with emergency incidents. We propose that two interpretation paths may generate the relationship between experience and learning. First, individuals have a more direct, in-depth perception when participating in, or observing, emergency events. Their cognition of first-aid events changes, and then, they may be more likely to acquire relevant knowledge to avoid corresponding risks than those who have not experienced such events (Trope and Liberman, 2010). Second, the emergency experience can trigger behaviors to help others manage current difficulties. Prosociality from watching urgent events can motivate people to learn skills for helping others (Penner et al., 2005).

## Literature Review

It has been proved that experience could motivate learning behavior (Liu et al., 2017). Further, the experience of emergencies can increase an individual's learning intention regarding relevant knowledge and skills (Hoffmann and Muttarak, 2017). As a predictive indicator of behavior, learning intention can reflect the subjective possibility that an individual will implement a certain behavior (Ajzen and Fishbein, 1980). Learning intention is usually defined as the degree of personal willingness or a plan to be put into action to narrow the gap between oneself and the desired goal through education and training (Kyndt et al., 2011).

We suggest that emergency experience may influence online first-aid learning intention in two ways. First, the relationship between emergency experience and learning intention is affected by the psychological distances (PDs) from emergency events. PDs guide people's behavior by changing their perception of objective things (Trope et al., 2007). For example, merchants can reduce customers' PDs from online shopping by uploading pictures of physical stores, thus improving purchase intention and encouraging first-time purchases (Darke et al., 2016). Next, the relationship between emergency experience and learning intention is affected by prosociality. The surrounding environment has an impact on the prosociality of individuals. More specifically, when people observed the prosocial behaviors of others, their prosociality may increase, and they are more likely to help others in the future (Krupka and Weber, 2009).

### Role of Psychological Distances

Different people's understanding of the same objective events varies because the PDs that people perceive differ (Liberman and Förster, 2009). According to the construal level theory, PDs are the degree to which an object is removed from the self, including dimensions of space distance, temporal distance, social distance, and uncertainty (Wakslak et al., 2006; Trope and Liberman, 2010). When a emergency event takes place in more remote place, occurs in the more distant future, happens to people less like oneself, and is less likely to occur, the event is more psychologically distant (Liu et al., 2017).

The literature has proved that subjective experiences are essential factors that affect PDs (Jackson, 1982; Ramachandran and Hirstein, 1997; Van Boven et al., 2010). For example, individuals' PDs could be reduced by the direct experience of climate change events, and then, PDs would increase their attention to climate change (Akerlof et al., 2013; McDonald et al., 2015). Moreover, the direct experience of driving pure electric vehicles reduces consumers' PDs from electric cars and improves their consumption intention (Skippon and Garwood, 2011).

Moreover, construal level theory holds that PDs change people's level of construction of events, which causes changes in their thoughts and behaviors (Trope et al., 2007). Individuals will use concrete, detailed, low-level words to construct events to which individuals are psychologically close. Conversely, they use abstract words to make them at a high level to represent distant events. (Liberman and Trope, 1998, 2008). For example, Spence et al. (2012) found that lower PDs made the public pay more attention to climate change and produced more avoidance behavior to mitigate the impact of climate change. In addition, the level of construction also affects psychological distances (Trope et al., 2007). For example, experiencing an emergency will make people pay attention to the low-level, detailed information of the emergency so that they will be more mentally close to the emergency.

### Role of Prosociality

Prosociality is an essential feature of humans correlated with cognition, emotion, and behavior to benefit others through social interaction (Zaki and Mitchell, 2013). People with prosociality tend to be more sensitive to the pain and needs of others, show more concern for others, and are more willing to help others (Caprara et al., 2012).

Various factors can affect an individual's daily life and thus cause them to exhibit various types of prosocial behaviors (Eisenberg and Fabes, 1998). Personal experience can affect prosociality (Vollhardt and Staub, 2011). Moreover, when people observe the prosocial behaviors of others, their prosociality would increase (Rand et al., 2012). Another study showed that the charity level of staff working in the high-level charity department of a charitable organization would increase by being affected by other staff (Christakis and Fowler, 2009).

Prosociality can provide people with internal support and guide their behavior, regardless of the motivation to ask for a return (Berman et al., 2015). Batson (2014) found that when bystanders saw others in trouble, prosociality would make them feel empathy and sympathy and act to help others. Thus, prosociality will encourage the public to learn and use helpful skills to benefit others (Penner et al., 2005).

## Hypotheses Development

The present study aims to understand how the emergency experience affects online first-aid learning intention. We suggest two ways in which emergency experience may influence online first-aid learning intention. One possible explanation is that emergency experience can improve the online first-aid learning intention through changing people's PDs from first-aid events. We also suggest that the relationship between emergency experience and learning intention is affected by prosociality.

The previous study has highlighted that experience would affect future avoidance behavior (Sönmez and Graefe, 1998). An existing study has also shown that experiencing emergencies increases the public's perceptions and risk awareness about crises to improve their willingness to learn relevant risk-aversion skills (Hoffmann and Muttarak, 2017). Learning relevant risk avoidance knowledge and skills is an effective risk avoidance behavior, which can enhance people's ability to respond to similar incidents in the future and reduce the incidence of sudden death. First-aid learning, which is one of the specific behaviors to avoid risks in the future, requires further understanding and combines theory and practice. The observation of an emergency event will undoubtedly enhance the public's perception of the crisis and cognition of the event, which will improve the public's willingness to learn first aid. Therefore, we suggest that emergency experience can increase people's online first-aid learning intention. According to this argument, we propose the first hypothesis:

*H1: Emergency experience has a positive impact on online first-aid learning intention*.

Previous studies have found that experiencing a sudden disaster affects people's risk avoidance behavior by decreasing their PDs from risk (Spence et al., 2012). Therefore, we suggest that PDs mediate the effect of emergency experience on online first-aid learning intention. Experiencing an emergency means that the distance between the individual and the event is changed in the four dimensions of temporal distance, space distance, social distance, and possibility. The PDs between the public and the emergency event are reduced. Therefore, experiencing emergency events may affect individuals' PDs from emergency events. Previous studies have also shown that personal experience directly affects people's PDs from objective things (Van Boven et al., 2010).

At the same time, according to the construal level theory, changes in PDs could alter people's construal level of exact items and ultimately reflect in their thoughts and behaviors (Trope et al., 2007). For individuals, emergency experience changes the temporal distance, space distance, possibility, and social distance of emergency events which means that the emergency experience reduces the psychological distances. With the narrowing of psychological distances, individuals become more sensitive to risk events and are more willing to take corresponding measures to prevent such occurrences. Learning first-aid knowledge and skills is one of the specific behaviors for preventive measures. Therefore, the PDs of individuals to first aid events affects the willingness to learn first aid knowledge and skills. Given the hypothesis that the emergency experience affects PDs, and that PDs affect learning intention, it is reasonable to suppose that PDs play a mediating role in the relationship between the emergency experience and online first-aid learning intention, which leads to our second hypothesis:

*H2: Individual PDs mediates the relationship between their emergency experience and online first-aid learning intention*.

We also suggest that the influence of emergency experience on online first-aid learning intention can be mediated by prosociality. Prosocial behavior is voluntary, proactive, and beneficial to others (Zaki and Mitchell, 2013). Examples of such behaviors are caregiving, mutual coordination, donations, and learning to help others (Eisenberg and Miller, 1987). Previous studies have also shown that prosocial individuals are more willing to spend time and energy helping others than individualists (De Cremer and Van Lange, 2001). It is reasonable to infer that prosociality will encourage individuals to acquire some knowledge and skills helpful to others. Therefore, we believe that prosociality may affect the willingness of the public to learn first aid knowledge and skills. At the same time, prosocial tendencies will be affected by environmental factors (Hastings et al., 2007). When individuals engage in prosocial behavior in daily life or observe the prosocial behavior of others, prosociality can be strengthened (Gentile et al., 2009). To sum up, past experience of emergencies can influence prosociality to some extent. Simultaneously, prosociality can provide people with internal support and guide their behavior (Berman et al., 2015). Therefore, we test another mediation model, assuming that prosociality would mediate the relationship between emergency experience and willingness to learn first aid online:

*H3: Individuals' prosociality mediates the relationship between their emergency experience and online first-aid learning intention*.

## Materials and Methods

### Research Process

In October 2020, we conducted a survey based on a well-known domestic emergency Sina Weibo account (*Jizhenyeying*) in China that emergency department physicians established in 2012. These physicians shared concise, helpful, evidence-based guidelines on emergency treatment science articles and made videos based on rich first-aid experience. This account is dedicated to promoting reliable and practical first-aid knowledge and skills. In addition, it also provides online first-aid training courses with a mixture of first-aid theories and practices. The number of followers of this account exceeded 1.9 million at the end of November 2019.

The current cross-sectional survey used online questionnaires distributed through the official emergency account. All participants voluntarily participated in the study and gave written informed consent after completing the questionnaire. We informed them that all their answers would remain anonymous and confidential, and they could drop out at any time. The time required to answer all research questions is ~7 mins. The design of the research protocol followed the specific ethical requirements of the authors' college.

### Participants

We collected data from 804 adults using an online survey platform. We first excluded 456 samples who had studied first-aid knowledge and skills before. Subsequently, we excluded 29 samples aged above 45 years because older people are less able to learn online (Chu, 2010). Then, we used data from 319 adults. The age range of the respondents in this study was 18–44 years. They were from 98 cities of China, including all 21 megacities with a population of more than 10 million and 45 major cities across the country; 18 overseas Chinese participated from 11 different countries. The sample selection criterion was that each participant had paid attention to emergency events from online channels. The test sample demographic is shown in Table 1.

**Table 1:** Test sample demographic (*n* = 319).

**Demographic**	**Category**	***N***	**%**
Age	25 and younger	69	21.6
	25–34	159	49.8
	35–44	91	28.5
Gender	Female	76	23.8
	Male	243	76.2
Education level	Middle school and below	0	0.0
	High school	21	6.6
	College and university	238	74.6
	Master	55	17.2
	Doctor and above	5	1.6

### Research Tool

The independent variable of this study was the emergency experience, formed by the answers given to the following question: “How many emergency events have you experienced?” We measured the degree of first-aid experience of participants by the number of emergency experience they filled in.

The PDs scale consists of four PDs dimensions: temporal distance, space distance, social distance, and possibility (Spence et al., 2012). All the items were measured on a 5-point Likert scale, ranging from 1 to 5. For example, “My living area will less likely suffer emergency events (including accidents and acute diseases).” The higher the score, the nearer the PDs reported by the participants. (See Table 2).

**Table 2:** Likert scale of PDs.

**Questions**	**Scale**				
	**5**	**4**	**3**	**2**	**1**
**Likert scale of PDs**					
1. succnsimMy living area is likely to suffer emergency events (including accidents and acute diseases)	Strongly agree	Agree	Normal	Disagree	Strongly disagree
2. Emergency events is likely to have a big impact on me.	Strongly agree	Agree	Normal	Disagree	Strongly disagree
3. When, if at all, do you think you will suffer the emergency events?	We are already feeling the effects	One month later	Half year later	Several years later	Never
4. I am uncertain that emergency events is really happening	Strongly disagree	Disagree	Normal	Agree	Strongly agree

The prosociality scale consists of seven items, measured using the Likert 5-point scale, with the scores ranging from 1 (strongly disagree) to 5 (strongly agree). For example, “I am pleased to help my friends/colleagues in their activities.” Cronbach's alpha indicates good internal consistency (α= 0.896). The higher the score, the higher the prosociality reported by the participant. (See Table 3).

**Table 3:** Likert scale of prosocial.

**Questions**	**5**	**4**	**3**	**2**	**1**
**Likert scale of prosocial**					
1. I am pleased to help my friends/colleagues in their activities	Strongly agree	Agree	Normal	Disagree	Strongly disagree
2. I share the things that I have with my friends	Strongly agree	Agree	Normal	Disagree	Strongly disagree
3. I try to help others	Strongly agree	Agree	Normal	Disagree	Strongly disagree
4. I am available for volunteer activities to help those who are in need	Strongly agree	Agree	Normal	Disagree	Strongly disagree
5. I am emphatic with those who are in need	Strongly agree	Agree	Normal	Disagree	Strongly disagree
6. I am willing to make my knowledge and abilities available to others	Strongly agree	Agree	Normal	Disagree	Strongly disagree
7. I try to be close to and take care of those who are in need	Strongly agree	Agree	Normal	Disagree	Strongly disagree

Last, our dependent variable is online first-aid learning intention, which consisted of answers to the following four questions: “I intend to look for information about first-aid courses and learning activities in which I could participate”; “I intend to participate in a first-aid learning activity within the next year”; “Sometimes, I think about following a first-aid training within the next year”; “I intend to talk with persons in my surroundings about first-aid courses or pieces of training that I could follow”. Participants answered on a 5-point Likert scale ranging from 1 (strongly disagree) to 5 (strongly agree). The higher the score, the higher the participant's willingness to learn.

The demographic scale assessed the participants' age, gender, and education level. All tools were first pre-tested on a similar sample of adults (N = 32), and no difficulties were reported related to the items used in the questionnaire. We used SmartPLS 3 to analyze our data.

## Results

The mean value, standard deviation, and correlations of the main variables are shown in Table 4.

**Table 4:** Descriptive statistics and Pearson correlation matrix.

**Variable**	**Mean**	**SD**	**Cronbach's α**	**Variable**						
				**C1**	**C2**	**C3**	**Exp**	**PDs**	**Pro**	**LI**
C1	2.069	0.705	–	1	–	–	–	–	–	–
C2	2.138	0.531	–	0.176	1	–	–	–	–	–
C3	1.762	0.426	–	0.086	0.159	1	–	–	–	–
Exp	0.245	0.551	–	0.029	0.035	−0.072	1	–	–	–
PDs	3.843	2.171	–	−0.070	−0.089	−0.068	−0.574	–	–	–
Pro	2.765	5.016	0.896	0.026	0.063	0.097	0.084	−0.277	0.760	–
LI	2.924	3.645	0.850	0.073	0.073	0.172	0.103	−0.237	0.450	0.830

*C1, C2, and C3 refer to three control variables: age, education, gender; Exp, PDs, Pro and LI refer to emergency experience, psychological distances, prosociality, and online first-aid learning intention*.

This study explored the mediating effects of PDs and prosociality on the relationship between emergency experience and learning intention. The total influence of emergency experience on online first-aid learning intention (without considering mediating factors) was significant [path coefficient = 0.112, *t* = 2.523, *p* <0.01, SE = 0.044, 95% CI (0.052, 0.208)], supporting Hypothesis 1.

**Figure 1:**
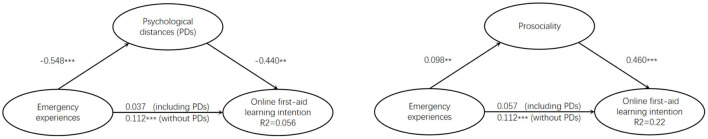
Psychological distances (PDs) and prosociality as mediators between emergency experiences and online first-aid learning intention.

First, we tested the relationship between emergency experience and PDs, and the relationship between PDs and online first-aid learning intention without the mediating role of PDs. Emergency experience also had a significant effect on PDs [path coefficient = −0.571, *t* = 10.689, *p* <0.01, SE = 0.053, 95% CI (−0.677, −0.467)]. In addition, PDs and learning intention were significantly positively correlated [path coefficient = −0.440, *t* = 9.830, *p* <0.01, SE = 0.045, 95% CI (−0.539, −0.362)], indicating that individuals with smaller PDs are more likely to have online first-aid learning intention. In the model with the mediator of PDs, PDs was a significant predictor of online first-aid learning intention [path coefficient = −0.255, *t* = 2.035, *p* <0.05, SE = 0.125, 95% CI (−0.478, −0.026)]. The direct effect of emergency experience on learning intention was insignificant [path coefficient = 0.037, *t* = 1.107, *p* = 0.268, SE = 0.034, 95% CI (−0.029, 0.103)]. However, the indirect effect of emergency experience on learning intention through PDs was significant, suggesting the relationship between emergency experience and learning intention is mediated by PDs, supporting Hypothesis 2. Figure 1 shows the mediation model that contains the values of the normalized coefficients for each relationship between variables.

Similarly, we found a significant correlation between emergency experience, prosociality, and learning intention. More specifically, higher emergency experience was associated with higher prosociality levels [path coefficient = 0.103, *t* = 2.078, *p* <0.05, SE = 0.050, 95% CI (−0.166, 0.1622)]. In addition, prosociality and online first-aid learning intention were significantly positively correlated [path coefficient = 0.467, *t* = 9.125, *p* <0.01, SE = 0.051, 95% CI (0.359, 0.558)], indicating that individuals with higher prosociality are more willing to learn. In the model with prosociality as the mediator, prosociality was a significant predictor of online first-aid learning intention [path coefficient = 0.460, *t* = 8.776, *p* <0.01, SE = 0.052, 95%CI (0.361, 0.566)]. The direct effect of emergency experience on learning intention was insignificant [path coefficient = 0.057, *t* = 1.541, *p* = 0.123, SE = 0.037, 95% CI (−0.018, 0.128)]. However, the indirect effect of emergency experience on learning intention through prosociality was significant, suggesting that the relationship between emergency experience and learning intention is fully mediated by prosociality. Thus, Hypothesis 3 was supported. Figure 1 shows the mediation model that contains the values of the normalized coefficients for each relationship between variables.

Additionally, we conducted a bootstrap analysis to estimate the mediating effect with 319 samples (Preacher et al., 2007; SPSS Process Macro Model 4). For hypothesis 2, the covariates were the same as the results showed in Table 2, and the results of the mediating effect was: path coefficient = 0.140, *t* = 2.266, *p* = 0.024, SE = 0.062, 95% CI (−0.067, 0.225). For hypothesis 3, the covariates were also the same with the results showed above, and the result of mediating effects was: path coefficient = 0.045, *t* = 1.899, *p* = 0.058, SE = 0.024, 95% CI (−0.003, 0.092).

At last, we performed a sensitivity analysis G*Power 3.1 to examine the statistical power of this article (Faul et al., 2007). For hypothesis 2, the size of the smallest effect in our model requested Cohen's f 2 = 0.43. With an alpha = 0.05, power = 0.9, and sample of 319, the smallest f 2 requested 0.25. For hypothesis 3, the size of the smallest effect requested Cohen's f 2 = 0.27. With an alpha = 0.05, power = 0.9, and sample of 319, the smallest f 2 requested 0.26. All the effects were above the value of the predicted minimal detectable effect size, which suggests that this study is appropriately powered.

## Discussion

As expected, emergency experience and online first-aid learning intention had a significant and positive relationship. In addition, we found that PDs and prosociality played mediating roles in the relationship between emergency experience and online first-aid learning intention.

First, the results showed that emergency experience could significantly predict online first-aid learning intention. Fewer studies have directly examined this relationship. However, existing studies have shown that experiencing a disaster may increase individuals' awareness about the potential for destruction and enhance their intention of acquiring disaster-related knowledge and skills to cope with subsequent disaster threats (Sattler et al., 2000).

Second, the results suggested that emergency experience affected online first-aid learning intention through the mediating role of PDs. The findings in this study indicated that emergency experience significantly predicted PDs. In line with existing studies, perceptions of PDs could be influenced by subjective experience (Jackson, 1982), which experience may generate. Meanwhile, the findings in this study suggested that PDs are significantly associated with online first-aid learning intention. Other studies have already found that PDs may influence subsequent behaviors. For example, PDs from climate change could predict people's willingness to engage in pro-environmental conduct (Wang et al., 2019). Further, this study found a significant mediating role of PDs in the relationship between emergency experience and online first-aid learning intention.

Last, the results also showed the mediation path of prosociality between emergency experience and online first-aid learning intention. We confirmed that prosociality was positively associated with the emergency experience. Studies have found that when people observe prosocial behavior in others, their prosocial tendencies may increase (Van Baaren et al., 2004). As the literature has already indicated, prosociality can provide people with internal support and guide their behavior, such as learning behavior (Berman et al., 2015), regardless of the motivation to ask for a return. Our findings confirm that prosociality is positively associated with online first-aid learning intention. People experience emergency events and observe others' prosocial behaviors, which can strengthen prosociality and increase people's tendency to learn first-aid knowledge and skills to help others.

## Conclusion

This study investigated how experiencing an emergency influenced online first-aid learning intention and found support for all the hypotheses.

Firstly, we found that emergency experience significantly predicted online first-aid learning intention without mediators, enriching relevant research on the influencing factors of online first-aid learning intention.

Secondly, this article verified the mediating role of PDs and prosociality in this relationship, expanding the theoretical analysis of the factors affecting online first-aid learning intention. On the one hand, based on construal level theory, the influence of emergency experience on online first-aid learning intention through PDs is explored, and the application of construal level theory is extended. The emergency experience greatly affected the psychological distances from emergency events, and the PDs of emergency events significantly influenced online first-aid learning intention. However, emergency experience had no significant direct influence on online first-aid learning intention on adding the mediator of the PDs of emergency events, illustrating the full-mediation effect of the PDs. On the other hand, the conclusions of this article also extend the application of prosocial. The results showed that emergency experience significantly affected prosociality and greatly influenced online first-aid learning intention. Meanwhile, emergency experience had no significant direct influence on online first-aid learning intention when adding the mediator, illustrating a full-mediation effect of prosociality.

### Limitations and Future Research

Although this study yielded significant research findings, it has certain limitations. First, we collected data from a well-known domestic emergency account. We focused on promoting the online first-aid learning intention of individuals who were concerned about emergency care. However, people within China generally lack the knowledge and skills related to first aid and are unaware of the importance of acquiring first-aid knowledge and skills. Therefore, future research can be conducted to promote the first-aid learning intention of the general public. Second, since the online learning ability of older people is relatively weak, we focused on those <45 years old. Future research can also expand the study of online first-aid learning intention by including middle-aged and older people.

## Data Availability Statement

The raw data supporting the conclusions of this article will be made available by the authors, without undue reservation.

## Author Contributions

SZ: acquisition of data, conception and design of study, analysis and interpretation of data, drafting the manuscript, and approve final paper for publication. HG: acquisition of data, conception and design of study, drafting the manuscript, and approve final paper for publication. XJ: revise the manuscript critically for important intellectual content and approve final paper for publication JM: acquisition of data and approve final paper for publication. All authors contributed to the article and approved the submitted version.

## Conflict of Interest

The authors declare that the research was conducted in the absence of any commercial or financial relationships that could be construed as a potential conflict of interest.

## Publisher's Note

All claims expressed in this article are solely those of the authors and do not necessarily represent those of their affiliated organizations, or those of the publisher, the editors and the reviewers. Any product that may be evaluated in this article, or claim that may be made by its manufacturer, is not guaranteed or endorsed by the publisher.
